# Tumor-biopsy stratification based on mTOR-pathway activity and functional mutations in the upstream genes *PIK3CA* and *PTEN*

**DOI:** 10.18632/oncotarget.21348

**Published:** 2017-09-28

**Authors:** Jean-François Laes, Sebastien Sauvage, Gregori Ghitti

**Affiliations:** ^1^ OncoDNA SA, 6041 Gosselies, Belgium

**Keywords:** cancer, biomarker, tumor stratification, mTor activation, PIK3CA activation and PTEN loss

## Abstract

The mechanistic target of the rapamycin (mTOR) pathway is frequently activated in human cancers. Our objective was to evaluate relationships between mTOR-pathway activity and functional mutations in the upstream genes *PIK3CA* and *PTEN* in solid-tumor biopsies from a broad selection of cancer types.

Formalin-fixed paraffin-embedded (FFPE) tumor samples were analyzed by immunohistochemistry (IHC) and next-generation sequencing (NGS). TOR-pathway activation was identified by expression (by IHC) of the downstream effector p-4E-BP1. Activating *PIK3CA* mutations and null *PTEN* mutations were identified by NGS, and for *PTEN*, confirmed by IHC.

Overall, mTOR-pathway activation was identified in 444/538 (83%) samples representing 40 different cancer types. Functional mutations in either or both *PIK3CA* and *PTEN* genes were identified in 173/538 (32%) samples. *PIK3CA* mutations were identified in 60/538 (11%) samples, *PTEN* mutations were identified in 155/538 (29%) samples and mutations in both *PIK3CA* and *PTEN* were identified in 18/538 (3%) samples. Overall, mTOR-pathway activation was not significantly associated with the *PIK3CA* and *PTEN* genotypes. However, all 18 samples with both *PIK3CA* and *PTEN* mutations also displayed mTOR-pathway activation (χ^2^ *p*=0.0471). Also, out of a total of 95 breast cancer samples, there were 5 breast-cancer samples which did not have mTOR-pathway activation, and all 5 (100%) of these had *PIK3CA* and *PTEN* mutations compared to 51/90 (57%) in the breast-cancer samples with mTOR-pathway activation (*χ^2^* *p*=0.0134). Finally, the percentages of *PIK3CA* mutations were higher in colorectal-cancer samples which had mTOR-pathway activation (9/27, 33%) than in colorectal-cancer samples without mTOR-pathway activation (6/44; 14%; *χ^2^ p*=0.0484).

Therefore, tumor-biopsy analyses based on combined mTOR-pathway biomarkers (and combined NGS and IHC assessments) could potentially provide treatment-informative stratification for particular cancer types.

## INTRODUCTION

The mechanistic target of the rapamycin (mTOR) pathway is frequently activated in human cancers [1, 2]. The mTOR pathway acts as a sensor of the availability of metabolites and amino acids, and it regulates a wide range of cellular functions including cell growth, proliferation, and metabolism [1–3]. The protein 4E-BP1 (coded by *EIF4EBP1* gene) is a downstream effector of the mTOR pathway [1–4]. Protein synthesis is controlled by mTOR through direct phosphorylation of 4E-BP1 to p4E-BP1, and once phosphorylated, p4E-BP1 can no longer bind to eIF4F, a translation initiation factor. The 4E-BP1/eIF4E-BP1 complex regulates cell growth and proliferation, and p4E-BP1 was shown to be a prognostic marker in grade II-IV astroglial FFPE tumor samples obtained from 111 patients [5]. Indeed, high expression of p4E-BP1 has been associated with mTOR-pathway activation and cancer [1, 5–9].

The oncogene *PIK3CA* coding for the phosphatidylinositol 3-kinase (PI3K) p110α subunit and the tumor suppressor gene *PTEN* coding for the Phosphatase and Tensin Homolog lie upstream of the mTOR pathway. Activating mutations in *PIK3CA* or null mutations in *PTEN* and its loss of expression can lead to mTOR-pathway activation [1, 3, 10–12]. Certain inhibitors of mTOR and PI3K have been approved for the treatment of some types of cancer, and these and other inhibitors of mTOR and PI3K are under investigation in a variety of cancer settings [12–18]. Hence, stratification of tumor types by *PIK3CA* or *PTEN* mutations or expression, in combination with the mTOR activity status, could provide additional information concerning disease prognosis as well as potential sensitivity or resistance to cancer treatments.

The objective of this study was to evaluate the relationships between mTOR activity and the mutation status of the *PIK3CA* and *PTEN* genes. We conducted a prospective analysis of solid-tumor biopsies from a broad selection of cancer types. The activation of the mTOR pathway was determined by positive IHC-staining for p-4E-BP1. Activating mutations in *PIK3CA* and null mutations in *PTEN* were identified by NGS. Null mutations of *PTEN* (which could also have included potential epigenetic silencing) were confirmed by assessing loss of expression using IHC.

## RESULTS

In total, 538 samples representing 40 different cancer types were evaluated (Table 1). The three most frequently represented cancer types were colorectal cancer (71 samples), non-small-cell lung cancer (64 samples) and hormone receptor positive (HR+) breast cancer (61 samples). Fifteen cancer types were represented by 10 or more samples. In order to further explore the pathway, we also included analyses of samples from an additional 25 cancer types: 16 cancer types were represented by between 2-9 samples, and 9 cancer types were represented by single samples. Among all samples, no activating mutations in *mTOR*, *TSC1*, or *TSC2* genes were identified. No patients from whom the samples were derived were undergoing treatment with PI3K or mTOR inhibitors.

**Table 1:** Characterization of tumor biopsies by mTOR pathway activation and the presence of PIK3CA and PTEN functional mutations

Number of biopsy samples															
									mTOR active				mTOR inactive		
Tumor type	Total	mTOR active	mTOR inactive	%samples with mTOR active/total	*PIK3CA^GOF^* and/or *PTEN^LOF^*	*PIK3CA^WT^* and *PTEN^WT^*	% samples with at least one mutation/total	*PIK3CA^GOF^, PTEN*^LOF^	*PIK3CA^WT^, PTEN*^LOF^	*PIK3CA^GOF^, PTEN*^WT^	*PIK3CA^WT^, PTEN*^WT^	*PIK3CA^GOF^, PTEN*^LOF^	*PIK3CA^WT^, PTEN*^LOF^	*PIK3CA^GOF^, PTEN*^WT^	*PIK3CA^WT^, PTEN*^WT^
Overall	538	444	94	83	173	365	32	18	35	89	302	0	7	24	63
Colorectal cancer	71	44	27	62	23	48	32	0	4	6	34	0	4	9	14
Non-small-cell lung cancer	64	56	8	88	15	49	23	0	3	10	43	0	0	2	6
Breast cancer HR+	61	59	2	97	31	30	51	4	16	11	28	0	0	0	2
Ovarian cancer	54	46	8	85	13	41	24	2	2	7	35	0	1	1	6
Pancreatic cancer	33	25	8	76	3	30	9	0	0	2	23	0	0	1	7
Triple-negative breast cancer	27	26	1	96	18	9	67	5	6	7	8	0	0	0	1
Sarcoma	27	23	4	85	5	22	19	0	0	4	19	0	0	1	3
Cholangiocarcinoma	20	16	4	80	2	18	10	0	0	1	15	0	1	0	3
Gastric cancer	19	16	3	84	4	15	21	0	0	3	13	0	0	1	2
Endometrial carcinoma	17	15	2	88	10	7	59	4	0	4	7	0	1	1	0
Hepatocellular carcinoma	16	9	7	56	11	5	69	0	0	7	2	0	0	4	3
Glioblastoma	14	13	1	93	6	8	43	0	0	6	7	0	0	0	1
Kidney cancer	11	8	3	73	4	7	36	0	0	3	5	0	0	1	2
Melanoma	11	10	1	91	1	10	9	0	0	1	9	0	0	0	1
Prostate cancer	10	7	3	70	3	7	30	0	0	1	6	0	0	2	1
Cervix adenocarcinoma	9	8	1	89	2	7	22	0	0	2	6	0	0	0	1
Small-cell lung cancer	8	8	0	100	4	4	50	0	0	4	4	0	0	0	0
Breast cancer HER2+	7	5	2	71	2	5	29	2	0	0	3	0	0	0	2
Head and neck cancer	7	4	3	57	1	6	14	0	0	1	3	0	0	0	3
Urinary bladder cancer	6	5	1	83	4	2	67	1	1	1	2	0	0	1	0
Adenoid cystic carcinoma	5	5	0	100	1	4	20	0	0	1	4	0	0	0	0
Esophageal cancer	5	4	1	80	1	4	20	0	0	1	3	0	0	0	1
Salivary gland cancer	5	5	0	100	2	3	40	0	1	1	3	0	0	0	0
Leiomyosarcoma	4	3	1	75	1	3	25	0	0	1	2	0	0	0	1
Thymoma & thymic carcinoma	4	3	1	75	0	4	0	0	0	0	3	0	0	0	1
Astrocytoma	3	3	0	100	0	3	0	0	0	0	3	0	0	0	0
Tongue	3	3	0	100	0	3	0	0	0	0	3	0	0	0	0
Neuroendocrine tumors	2	2	0	100	1	1	50	0	0	1	1	0	0	0	0
Osteosarcoma	2	1	1	50	1	1	50	0	0	1	0	0	0	0	1
Penile cancer	2	2	0	100	0	2	0	0	0	0	2	0	0	0	0
Testicular cancer	2	2	0	100	0	2	0	0	0	0	2	0	0	0	0
Adnexal skin carcinoma	1	1	0	100	0	1	0	0	0	0	1	0	0	0	0
Anaplastic oligodendrogliomas	1	1	0	100	1	0	100	0	1	0	0	0	0	0	0
Duodenal adenocarcinoma	1	1	0	100	1	0	100	0	0	1	0	0	0	0	0
Medulloblastoma	1	1	0	100	1	0	100	0	1	0	0	0	0	0	0
Paraganglioma	1	0	1	0	0	1	0	0	0	0	0	0	0	0	1
Parathyroid cancer	1	1	0	100	1	0	100	0	0	1	0	0	0	0	0
Pineal astrocytoma	1	1	0	100	0	1	0	0	0	0	1	0	0	0	0
Primary peritoneal cancer	1	1	0	100	0	1	0	0	0	0	1	0	0	0	0
Thyroid cancer	1	1	0	100	0	1	0	0	0	0	1	0	0	0	0

GOF, gain-of-function mutation; and LOF, loss of function (null) mutation

mTOR-pathway activation was identified (by high expression of the downstream effector p-4E-BP1) in 444/538 (83%) samples (Table 1). Of the cancer types with 10 or more representative samples, mTOR-pathway activation was most prevalent in HR+ breast-cancer samples (59/61; 97%) and triple-negative (absence of estrogen and progesterone receptors and the absence of HER2 overexpression) breast-cancer samples (26/27, 96%); mTOR-pathway activation was least prevalent in hepatocellular-carcinoma samples (9/16; 56%) and colorectal-cancer samples (44/71; 62%).

Functional mutations in either or both *PIK3CA* (by NGS) and *PTEN* genes were identified (by NGS and by IHC) in 173/538 (32%) samples (Table 1). Activating mutations in *PIK3CA* gene were identified in 60/538 (11%) samples, null mutations in *PTEN* gene were identified in 155/538 (29%) samples and both activating mutations in *PIK3CA* and null mutations in *PTEN* genes were identified in 18/538 (3%) samples. Of the cancer types with 10 or more representative samples, *PIK3CA* and/or *PTEN* mutations were most prevalent in hepatocellular carcinoma samples (11/16; 69%), triple-negative breast-cancer samples (18/27, 67%), endometrial-carcinoma samples (10/17, 59%), and HR+ breast-cancer samples (31/61; 51%). *PIK3CA* and/or *PTEN* mutations were least prevalent in pancreatic-cancer samples (3/33; 9%) and melanoma samples (1/11; 9%). Mutations in both *PIK3CA* and *PTEN* genes were most prevalent in endometrial-carcinoma samples (4/17, 24%) and triple-negative breast-cancer samples (5/27, 19%).

Among the 444 samples with mTOR-pathway activation, 107/444 (24%) had activating mutations in the *PIK3CA* gene, 53/144 (12%) had null mutations in the *PTEN* gene, and 142/444 (32%) had mutations in either or both *PIK3CA* and *PTEN* genes (Table 1 and Table 2). For those 94 samples without mTOR-pathway activation, a similar proportion also had mutations in the *PIK3CA* gene (24/94; 26%; *χ^2^ p*=0.7687), in the *PTEN* gene (7/94; 7%; *χ^2^* *p*=0.2090), and in either the *PIK3CA* gene or the *PTEN* gene or both (31/94; 33%; *χ^2^* *p*=0.1753). Overall, mTOR-pathway activation was not significantly associated with the *PIK3CA* and *PTEN* genotypes.

**Table 2:** Contingency-table assessment of relationships between mTOR pathway activation and *PIK3CA* and *PTEN* genotypes

Population (Total number of biopsy samples)	Genotype	Number of biopsy samples		χ^2^ *p*-value
		mTOR-pathway status		
		Active (% active)	Inactive (% inactive)	
All (538)	*PIK3CA^GOF^*	107 (24)	24 (26)	0.769
	*PIK3CA*^WT^	337 (76)	70 (74)	
All (538)	*PTEN^LOF^*	53 (12)	7 (7)	0.209
	*PTEN^WT^*	391 (88)	87 (93)	
All (538)	*PIK3CA^GOF^* and/or *PTEN^LOF^*	142 (32)	31 (33)	0.851
	*PIK3CA^WT^/PTEN^WT^*	302 (68)	63 (67)	
All (538)	*PIK3CA^GOF^/PTEN^LOF^*	18 (4)	0 (0)	0.175
	*PIK3CA^GOF^/PTEN^WT^*	35 (8)	7 (7)	
	*PIK3CA^WT^/PTEN^LOF^*	89 (20)	24 (26)	
	*PIK3CA^WT^/PTEN^WT^*	302 (68)	63 (67)	
All (538)	*PIK3CA^GOF^/PTEN^LOF^*	18 (4)	0 (0)	0.047
	*PIK3CA^GOF^/PTEN^WT^*, or *PIK3CA^WT^/PTEN^LOF^*, or *PIK3CA^WT^/PTEN^WT^*	426 (96)	94 (100)	
All breast cancer (95)	*PIK3CA^GOF^* and/or *PTEN^LOF^*	51 (57)	0 (0)	0.013
	*PIK3CA^WT^/PTEN^WT^*	39 (43)	5 (100)	
Colorectal cancer (71)	*PIK3CA^GOF^*	6 (14)	9 (33)	0.048
	*PIK3CA^WT^*	38 (86)	18 (67)	

GOF, gain-of-function mutation; and LOF, loss of function (null) mutation

However, when considered as a separate category, all 18 samples with mutations in both *PIK3CA* and *PTEN* genes (breast cancer, endometrial carcinoma, ovarian cancer and urinary bladder cancer) also displayed mTOR-pathway activation (χ^2^ *p*=0.0471; Table 2). There were also notable potential relationships identified with certain cancer types. Out of a total of 95 breast cancer samples, 5 breast-cancer samples did not have mTOR-pathway activation, and all 5 (100%) of these had *PIK3CA* and *PTEN* mutations compared to 51/90 (57%) in the breast-cancer samples with mTOR-pathway activation (*χ*^2^ *p*=0.0134; Table 2). Finally, the percentages of *PIK3CA* mutations were higher in colorectal-cancer samples which had mTOR-pathway activation (9/27, 33%) than in colorectal-cancer samples without mTOR-pathway activation (6/44; 14%; *χ^2^ p*=0.0484; Table 2).

## DISCUSSION

This study found that a majority of solid tumors from a diverse range of cancer types display a potential aberrant activation of the mTOR pathway, and a minority of solid tumors contain functional mutations in the *PIK3CA* and *PTEN* genes (including for PTEN, a loss of expression also potentially related to epigenetic-silencing).

Although no overall association was identified between the activation of the mTOR pathway and the presence of functional mutations in the *PIK3CA* and *PTEN* genes, this may have been a reflection of differences between different cancer types within the limited sizes of those sample populations. Within particular categories and for particular cancer types, potential associations were identified. The combination of PIK3CA activating mutation and PTEN loss of function was associated with mTOR-pathway activation, most notably in the breast-cancer samples. This particular combination has been associated with poor outcomes to treatment with HER2/neu receptor inhibitor (trastuzumab) or PI3K inhibitor (BYL719) in breast cancer [19, 20]. Conversely, in the colorectal-cancer samples the *PIK3CA* activating mutation was more frequently associated low mTOR activity. Although the clinical relevance of this combination remains to be clarified in colorectal cancer, in ER+/HER2- breast cancer, the *PIK3CA* activating mutation/low mTORC1 signaling was associated with better clinical outcomes to adjuvant ER-modulator (tamoxifen) treatment [21].

Our results suggest that stratification of tumors using the combination of mTOR-pathway biomarkers (and combined NGS and IHC technologies in their assessment) is potentially more informative than using a single biomarker. Although in our study there was no information regarding treatment follow-up and none of the patients from whom we obtained samples had participated in clinical trials evaluating inhibitor PIK3CA or mTOR inhibitors, this form of tumor analysis could support stratification in a prospective clinical trial evaluating the clinical benefit of (dual) PIK3CA/mTOR treatments in comparison with current standard treatments. For example, in a diverse range of tumors, the *PIK3CA* H1047R mutation was suggested to be associated with a favorable clinical response to PI3K/AKT/mTOR inhibitors [17]. By contrast, only a minimal association was suggested between *PIK3CA* and clinical outcome in a retrospective analysis of breast-cancer samples from the BOLERO2 clinical trial [22].

Indeed, using a single biomarker is perhaps not the best study design. Our results obtained from the breast cancer samples, for example, suggest that stratification could be used to increase the toxicity-benefit cost of a treatment such as everolimus, where there is a clear need for clinical trials with improved sub-group analyses [23]. Also, the TAMRAD study provided preliminary evidence for an mTORC1 activation marker as a predictive factor for everolimus efficacy (time-to-progression) in advanced breast cancer [24]. These exploratory results suggest that everolimus efficacy might be positively correlated with late effectors of mTORC1 activation, Akt-independent mTORC1 activation, and inversely correlated with the PI3K/Akt/mTOR pathway. They also found that subgroups most likely to exhibit improved TTP with tamoxifen/everolimus therapy, compared with tamoxifen alone, were patients with high p4EBP1, low 4EBP1, low liver kinase B1, low pAkt, and low PI3K.

Indeed, stratified trials are needed to develop biomarkers that can predict response to mTOR inhibition as well as other breast cancer treatments, and our results suggest that the same suggestion for future stratification in clinical trials holds for a variety of cancer types, e.g. the several tumor sample types which had mutations in both *PIK3CA* and *PTEN* genes and also displayed mTOR-pathway activation, and the higher percentages of *PIK3CA* mutations that were observed in colorectal-cancer samples with than without mTOR-pathway activation.

Further analyses are needed to identify other key biomarkers and, ultimately, increase the predictive capacity of the stratification.

## MATERIALS AND METHODS

### Sample acquisition

All samples were derived from metastatic cancers from patients undergoing routine systemic therapies. Each sample was obtained as a FFPE block and satisfied the pathology entry criteria: tumor cellularity >10%; tumor section ≥4 mm^2^ with no necrosis and ≤20% lymphocyte infiltration. Tumors were macrodissected to remove contaminating normal tissue. Each patient provided informed consent for tumor-analysis data to be published.

### NGS and sequence-data processing

Sample preparation and sequencing was performed using an ISO15189-accredited process by OncoDNA, Gosselies, Belgium. Briefly, DNA was extracted from FFPE tissue using the Qiagen DNA FFPE Tissue Kit or Qiagen DNA Blood Mini Kit (Qiagen, Valencia, CA, USA). DNA quantity was measured using the Qubit 2.0 Fluorometer (Thermo Fisher Scientific, Waltham, MA, USA). A sequence library for a given sample was generated using the Ion AmpliSeq Library kit 2.0 (Thermo Fisher Scientific, Waltham MA, USA) in accordance with the manufacturer's instructions. A library was prepared from 10 ng or 50 ng DNA and the OncoDEEP primer panel or OncoDEEP Clinical primer panel, respectively (designed to amplify either 207 or >16452 amplicons covering either hotspot mutations or whole exons, respectively). The primers used in the amplification were partially digested by Pfu enzyme. The product of digestion was then ligated with corresponding barcoded adapters and purified using Ampure Beads (Beckman Coulter, Inc., Indianapolis, IN, USA). This purified product was amplified for 5 further cycles and re-purified using Ampure Beads to generate the library sample. The IonChef system (Thermo Fisher Scientific, Waltham, MA, USA) for emulsion PCR and chip loading was performed with 10 pmol library DNA per sample. The average coverage was set at 1000⊆ to be able to detect a minor allele frequency ≥5%. The Ion PGM, the Ion Proton or the 5XL system was used in accordance with the required throughput.

The sequence data were initially analyzed by using Torrent Suite software (Thermo Fisher Scientific, Waltham, MA, USA). Somatic mutations were identified using Variant Caller 4.0 software (Thermo Fisher Scientific, Waltham, MA, USA) using the somatic high-stringency parameters and hotspot pipelines. Candidate somatic mutations were further filtered based on, (1) coverage >100, (2) a forward-reverse ratio of 10%:90%, (3) occurrence in known protein-coding regions (i.e., missense, nonsense frameshift mutations or splice site alterations), and (4) the exclusion of intronic and silent mutations. A manual inspection step was also used to exclude potential artefactual changes. Protein-function-related mutations were distinguished from common germline mutations by reference to the dbSNP Human Variation Sets in VCF format, Version 138. The activating mutations in *PIK3CA* included those mutations that code for amino-acid substitutions E545, E546 and H1047. All known loss-of-function (null) mutations were considered for *PTEN*.

### Immunohistochemistry (IHC)

FFPE tumor samples were processed using standard procedures for IHC. PTEN and p-4E-BP1 were detected on 4 μm sections using rabbit anti-PTEN (Clone D4.3 XP) and rabbit anti-p-4E-BP1 (Thr37/46; clone 236B4) from Cell Signaling Technology, Danvers, MA, USA.

**Figure 1:**
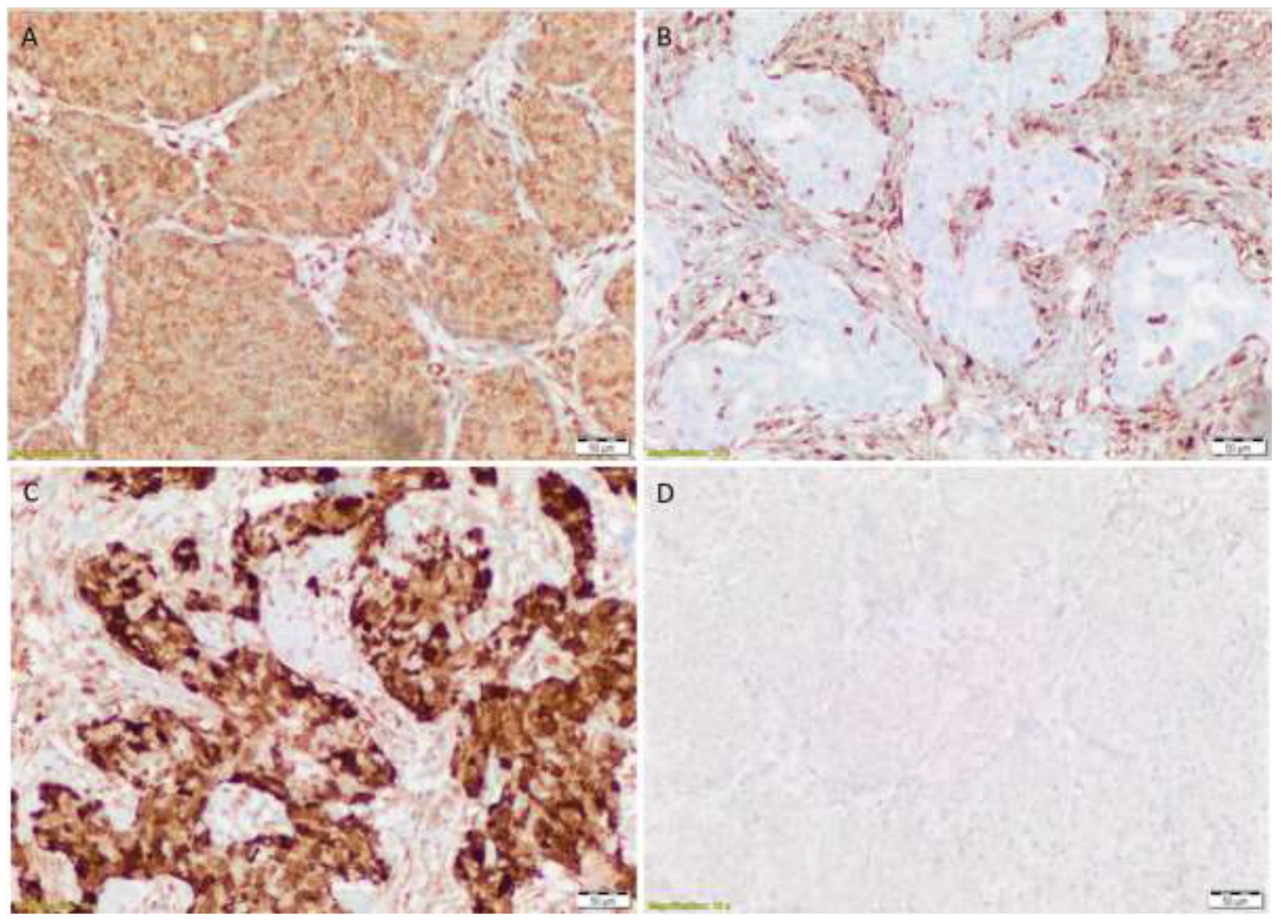
PTEN positive **(A)** PTEN negative **(B)** p4e-BP1 low **(C)** and p4E-BP1 high **(D)**.

Each slide was scored by two independent analysts who were blinded to the sample's description and to the other analyst's score of the slide. A consensus H-score (to define positive expression) was calculated in accordance with a predefined ISO15189-accredited method. Representative slides showing PTEN positive, PTEN negative, p4e-BP1 low, and p4E-BP1 high are shown in Figure 1. A positive stain was defined as an H-Score>100 (p-4E-BP1) and H-Score>10 (PTEN), in accordance with the following formulae:

H-Score (p-4E-BP1) = (%Cy^1+^+ %N^1+^)/2 + (%Cy^2+^+ %N^2+^) + 3⊆(%Cy^3+^+ %N^3+^)/2,

H-Score (PTEN) = %C^1+^ + 2⊆%C^2+^ + 3⊆%C^3+^,

where, %C, %Cy and %N are percentages of stained cells, cytoplasm and nuclei, respectively; and 1+, 2+ and 3+ indicate the staining intensities, low, moderate, and high, respectively.

### Statistics

The *χ^2^* test was used to evaluate the relationship between mTOR-pathway activation and *PIK3CA* and *PTEN* genotypes. No corrections were made with respect to multiple comparisons.
